# Mitogenome phylogenetics in the genus *Palaemon* (Crustacea: Decapoda) sheds light on species crypticism in the rockpool shrimp *P. elegans*

**DOI:** 10.1371/journal.pone.0237037

**Published:** 2020-08-18

**Authors:** Inés González-Castellano, Joan Pons, Enrique González-Ortegón, Andrés Martínez-Lage

**Affiliations:** 1 Departamento de Biología and Centro de Investigaciones Científicas Avanzadas (CICA), Universidade da Coruña, A Coruña, Spain; 2 Instituto Mediterráneo de Estudios Avanzados (IMEDEA), Consejo Superior de Investigaciones Científicas (CSIC) and Universitat de les Illes Balears, Esporles, Spain; 3 Instituto de Ciencias Marinas de Andalucía (ICMAN), Consejo Superior de Investigaciones Científicas (CSIC), Puerto Real, Spain; Shanghai Ocean University, CHINA

## Abstract

The genus *Palaemon* comprises worldwide marine and freshwater shrimps and prawns, and some of them are ecologically or commercially important species. *Palaemon* is not currently a monophyletic group, so phylogenetics and systematics are constantly changing. Species crypticism has been pointed out in several *Palaemon* species, being the clearest evidence in the European rockpool shrimp *P*. *elegans*. Here we sequenced and described seven European *Palaemon* mitochondrial genomes. The mitochondrial protein-coding genes were used, along with those of three other *Palaemon* species, to perform mitogenome phylogenetic analyses to clarify the evolutionary relationships within the genus, and particularly to shed light on the cryptic species found within *P*. *elegans*. The Messinian Salinity Crisis (5.3-5.9 Ma, late Miocene) was proposed to be the origin of this cryptic species and it was used as aged constraint for calibration analysis. We provide the largest and the first time-calibrated mitogenome phylogeny of the genus *Palaemon* and mitogenome substitution rate was estimated (1.59% per million years) in Decapoda for the first time. Our results highlighted the need for future systematics changes in *Palaemon* and crypticism in *P*. *elegans* was confirmed. Mitochondrial genome and *cox1* (1.41%) substitution rate estimates matched those published elsewhere, arguing that the Messinian Salinity Crisis was a plausible event driving the split between *P*. *elegans* and its cryptic species. Molecular dating suggested that Pleistocene glaciations were likely involved in the differentiation between the Atlantic and Mediterranean populations of *P*. *elegans*. On the contrary, the divergence between the Atlantic and Mediterranean populations of the common littoral shrimp *P*. *serratus* was greater and dated to be much older (4.5-12.3 Ma, Plio-Miocene), so we considered that they could represent two separated species. Therefore, species crypticism in the genus *Palaemon* seems to be a common phenomenon.

## Introduction

The genus *Palaemon* Weber, 1795 is the third most species-rich genus of the family Palaemonidae Rafinesque, 1815 [1,2], with marine, estuarine and freshwater shrimps and prawns worldwide distributed in tropical and temperate regions. *Palaemon* currently comprises 88 species, given the recent synonymization of the genera *Palaemonetes* Heller, 1869, *Coutierella* Sollaud, 1914 and *Exopalaemon* Holthuis, 1950 with *Palaemon* [2], and five new described species [3–6].

Species identification within this group is difficult due to their small size, morphological similarity and expressive plasticity of some characters used in species description, such as the number of teeth of the rostrum, the rostral shape or the position of the branchiostegal tooth [7]. Therefore, molecular data could be crucial to elucidate taxonomic ambiguities. Indeed, the family Palaemonidae has been the subject of numerous systematic and evolutionary studies due to its wide geographical distribution, large variety of habitats and the economic importance of some species [8]. Specifically in *Palaemon*, extensive worldwide phylogenetic analyses have been performed revealing the need for further systematic rearrangements. Two studies suggested to synonymise *Palaemonetes*, *Exopalaemon* and *Coutierella* with *Palaemon* since their paraphyly was demonstrated [9,10]. This new genus *Palaemon*, redefined by De Grave & Ashelby [2], mainly grouped species according to their biogeographical distribution. Nevertheless, the last phylogenetic assessment of the genus indicated that *Palaemon* clearly remained as a non-monophyletic group [8] where the species were divided into three different clades: one monospecific clade formed by *P*. *concinnus*; a second clade referred as *‘Alaocaris’* and composed by American species; and the larger *‘Palaemon’* clade that included *P*. *mercedae* as the sister species of the remaining species of the genus that would be *“Palaemon sensu stricto”*. Hence, phylogenetic relationships among *Palaemon* species are still a disputed systematic issue.

Regarding studies at the species level, phylogeographic analyses were conducted on several *Palaemon* species, detecting population structure in some of them that mainly corresponded to oceanographic discontinuities restricting gene flow. Presence of cryptic species was also pointed out in *P*. *atrinubes*, *P*. *debilis*, *P*. *serrifer* [9], *P*. *vulgaris* [11] and *P*. *elegans* [12,13]. Cryptic species occur when two or more genetically distinct molecular species (entities) were classified as a single nominal species because they are morphologically similar or indistinguishable [14,15]. Crypticism is particularly prone in the marine environment [16] and molecular markers have indicated the occurrence of cryptic species in a wide variety of marine taxa [e.g. 17–20]. Among the aforementioned species, the clearest evidence for the existence of a cryptic species was found in *P*. *elegans*, which has been the focus of our previous research.

The rockpool shrimp *P*. *elegans* Rathke, 1837 is considered an important species within the European coastline fauna due to its broad ecological niche and its wide and ongoing geographic expansion. Reuschel *et al*. [12] carried out a phylogeographic analysis in *P*. *elegans* using two mitochondrial DNA (mtDNA) markers. In that study, some individuals from the Mediterranean Sea exhibited highly divergent and diagnostic haplotypes relative to the rest of the individuals sampled in Atlantic and Mediterranean localities. They were named *P*. *elegans* type III and proposed to belong to a putative cryptic species. This genetic pattern was supported in later phylogeographic studies in the Mediterranean Sea also using mtDNA data [21,22]. Very recently, we have developed polymorphic microsatellite loci for this species [23] and accomplished a large analysis along its native distribution range that corroborated two clearly genetically distinct groups within *P*. *elegans* [13]. Our work strongly supported the existence of a cryptic and sympatric species within *P*. *elegans* in the Mediterranean Sea, providing nuclear evidence for the divergence signatures previously detected at mitochondrial level. The main question to be solved is the origin of the cryptic species. The desiccation of the Mediterranean Sea (Messinian Salinity Crisis 5.33-5.96 Ma; [24]) was postulated as the most likely scenario for the origin of this cryptic species [12,13], but unfortunately there are no known fossils of Palaemonidae near to that time.

Accordingly, the aim of the present study was to contribute for a better understanding of the phylogenetic relationships of the genus *Palaemon* and at the same time to confirm the cryptic species found within *P*. *elegans* in a phylogenetic framework. For this purpose, we revisited the phylogeny of the genus *Palaemon*, including *P*. *elegans*, using mitochondrial genome (mitogenome) sequences. Mitogenomes have been widely used to address phylogenetic questions in various groups, such as crustaceans [e.g. 25–27], thanks to high-throughput sequencing-based approaches that enable a fast and cost-effective bioinformatic mitogenome recovery from full genome data at low coverage [28]. Most metazoan mitogenomes are circular double stranded DNA molecules of about 14-18 kb in length, typically containing 13 protein-coding genes (PCGs), 2 ribosomal RNAs (12S rRNA and 16S rRNA) genes and 22 transfer RNAs (tRNAs) genes, as well as a large AT-rich non-coding control region [29,30]. As for *Palaemon*, the complete mitogenome of seven species has been characterized to date. Here we sequenced, assembled and annotated the mitogenome sequence of ten *Palaemon* specimens from seven species to conduct further phylogenetic analyses. To the best of our knowledge, this work provides the largest mitogenome phylogeny of the genus *Palaemon*, describing the mitogenome of six *Palaemon* species for the first time. The molecular clock for the mitochondrial protein coding genes in *Palaemon* was estimated by constraining the most recent common ancestor (mrca) of *P*. *elegans*, including the cryptic species, to the age of the Messinian Salinity Crisis. A rate estimate similar to that reported in other crustacean and other invertebrate species for similar data would be an advocacy of our hypothesis.

## Material and methods

### Ethics statement

No specific permits were required for shrimp collection and experimental work as they are unregulated marine invertebrates. The sampling localities were not privately owned or natural protected areas and neither endangered nor protected species were involved in the study.

### Sample collection, DNA extraction, library preparation and sequencing

**Fig 1:**
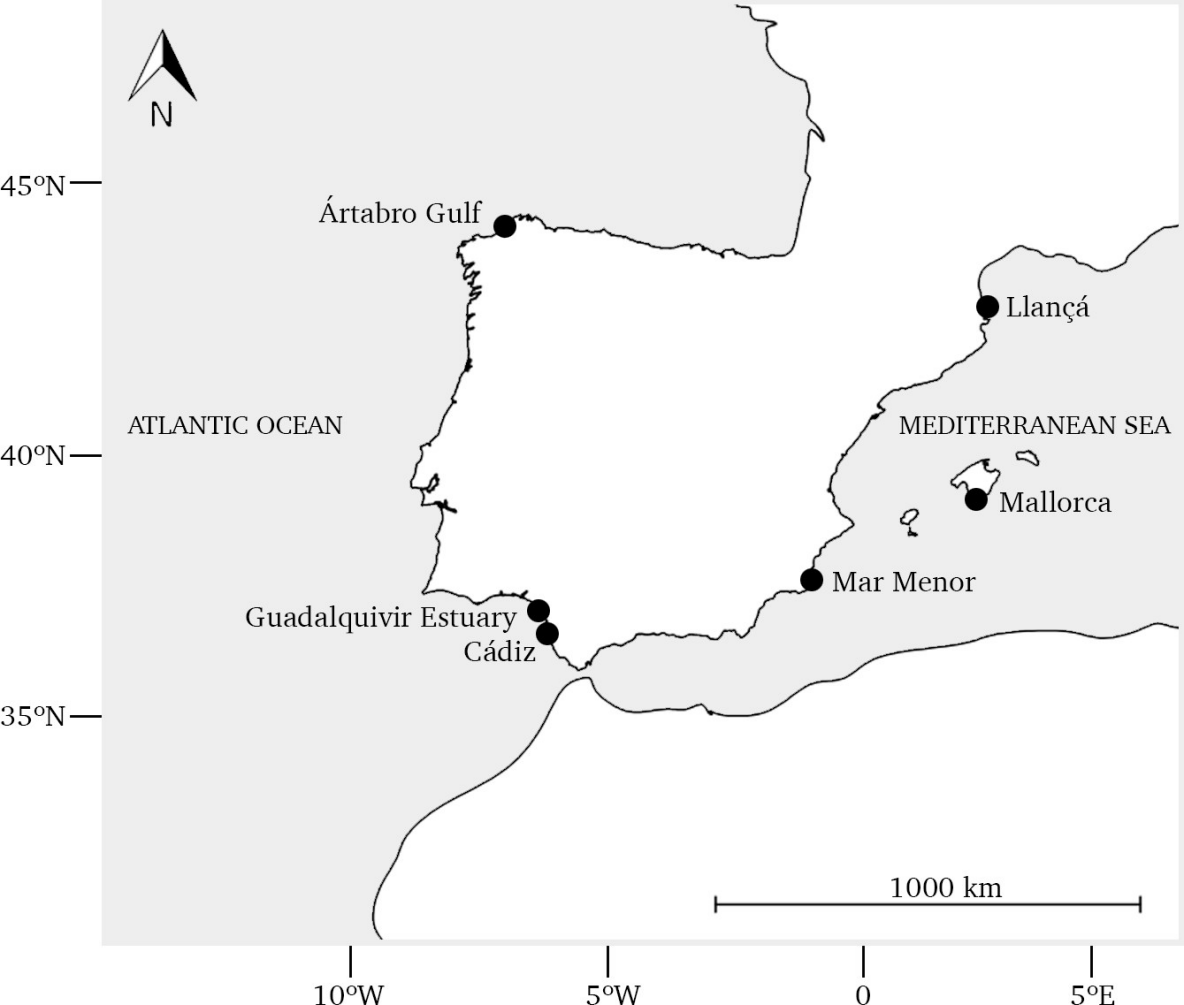
Geographical location of the sampling sites along the Atlantic and Mediterranean coast of the Iberian Peninsula. For labelling, see Table 1.

Ten specimens belonging to seven different species of the genus *Palaemon* were collected inshore between 2012 and 2019 from different sampling sites in Spain (Fig 1 and Table 1). One individual was collected for each species, except for *P*. *elegans* and for *P*. *serratus* for which three and two individuals were sampled, respectively, from Atlantic and Mediterranean localities since cryptic species may be present within them. Animals were captured with a fish trap and stored at laboratory in ethanol 96%. Morphological identification was based on key diagnostic features described in González-Ortegón & Cuesta [31]. Genomic DNA was extracted from 25 mg of abdominal muscle tissue using the NZY Tissue gDNA Isolation kit (NZYTech, Lisbon, Portugal) following the manufacturer’s instructions. DNA quality and concentration was determined with the NanoDrop ND-1000 spectrophotometer (Thermo Fisher Scientific, Waltham, USA). Voucher specimens and DNA aliquots were deposited at University of A Coruña (Spain). One library was prepared for each genomic DNA sample at AllGenetics & Biology SL (A Coruña, Spain) using the Nextera XT DNA Library Prep kit (Illumina, San Diego, USA) and sequenced in an Illumina NovaSeq 6000 platform (2x150 PE), yielding about 10 GB data (10-14 million reads) for each sample.

**Table 1:** Collecting samples details.

Species	Locality	Coordinates
***P*. *adspersus***	Atlantic Ocean: Gualdalquivir Estuary (Spain)	36°51'29"N 6°21'02"W
***P*. *elegans***	Atlantic Ocean: Ártabro Gulf (Spain)	43°18'48"N 8°33'51"W
***P*. *elegans***	Mediterranean Sea: Llançá (Spain)	42°21'06"N 3°11'15"E
***P*. *elegans* (cryptic species)**	Mediterranean Sea: Mallorca (Spain)	39°21'08"N 2°58'26"E
***P*. *longirostris***	Atlantic Ocean: Guadalquivir Estuary (Spain)	36°51'29"N 6°21'02"W
***P*. *macrodactylus***	Atlantic Ocean: Guadalquivir Estuary (Spain)	36°51'29"N 6°21'02"W
***P*. *serratus***	Atlantic Ocean: Ártabro Gulf (Spain)	43°18'48"N 8°33'51"W
***P*. *serratus***	Mediterranean Sea: Mallorca (Spain)	39°21'08"N 2°58'26"E
***P*. *varians***	Atlantic Ocean: Cádiz (Spain)	36°39'22"N 6°24'10"W
***P*. *zariquieyi***	Mediterranean Sea: Mar Menor (Spain)	37°39'38"N 0°48'24"W

### Mitochondrial genomes assembly and annotation

Reads were demultiplexed by specimens and transformed to fastq format using the software bcl2fastq2 (Illumina, San Diego, USA) as libraries were constructed with specimen-specific barcode sequences. Adapters and low-quality bases were removed with Trimmomatic v0.35 [32] using a sliding window of 6 bases and quality threshold below 20. Trimmed reads shorter than 100 bp were discarded. Quality control of raw and trimmed reads was performed using FastQC v0.11.5 [33]. Mitogenomes were *de novo* assembled from trimmed reads using SPAdes v3.9.0 [34] with kmer sizes of 21 and 35. Mitochondrial contigs were identified by BLASTn [35] against a mitochondrial genome sequence of *Palaemon annandalei* (NC_038117). Alternatively, if *de novo* assembly did not retrieved a mitochondrial contig with an expected size, mitogenomes were obtained using Geneious v8.0.5 [36] by iterative mapping of the trimmed reads on the mitochondrial genome of *Palaemon adspersus*, which was assembled as complete with SPAdes. The consensus sequence of each mitochondrial contig of about 17 Kb was mapped with trimmed reads to extend its sequence. Then, we run BLASTn searches using the python script circularizationCheck.py available in mitoMaker [37] to find matching 5’ and 3’ ends. This step corroborates the presence of overlapping ends in the consensus sequence, and after removing one identical end, the mitogenome is complete and circular. Mitogenomes were annotated using the MITOS2 Web Server [38] applying the invertebrate mitochondrial genetic code. Start and stop codon regions of PCGs and the 5′ and 3′ ends of ribosomal genes were manually refined by aligning to other *Palaemon* sequences available in GenBank (see below) using Muscle v3.8.1551 [39] implemented in SeaView v4.6.4 [40]. tRNA genes were detected and their secondary structures were inferred using MITOS2 with default settings, and 5’ and 3’ ends were validated by aligning orthologous sequences across species. Sequences of *trnS1* were manually curated because they included a short and unrealible dihydroxyuridine (DHU) arm in some species. Circular mitogenome plots were drawn using the OGDRAW online tool [41].

## Phylogenetic analyses

Concatenated sequences of the 13 PCGs of the newly obtained mitogenomes were used to conduct phylogenetic analyses in species of the genus *Palaemon*. The mitogenomes of seven *Palaemon* species reported elsewhere were also included in the analyses: *P*. *annandalei* (NC_038117), *P*. *capensis* (NC_039373), *P*. *carinicauda* (NC_012566), *P*. *gravieri* (NC_029240), *P*. *modestus* (MF687349), *P*. *serenus* (NC_027601) and *P*. *sinensis* (MH880828). Additionally, the mitogenomes of four species of the genus *Macrobrachium* (*M*. *rosenbergii* NC_006880, *M*. *lanchesteri* NC_012217, *M*. *bullatum* NC_027602, and *M*. *nipponense* NC_015073) were used as outgroups, since they belonged to the only genus of the familiy Palaemonidae with available mitogenomes in NCBI besides *Palaemon*. PCG sequences were first translated into proteins, aligned with Muscle with default parameters and back-translated to nucleotides conserving the integrity of codon triplets but excluding terminal stop codons. The 13 separate alignments (one per each PCG) were concatenated using Phyutility [42] into a single large dataset.

Phylogenetic analyses were conducted under Maximum Likelihood (ML) and Bayesian Inference (BI) frameworks using IQ-TREE v1.6.12 [43] and BEAST v1.10.4 [44], respectively. Best partition scheme and nucleotide substitution models were estimated in IQ-TREE using the command –sp TESTMERGE with 39 initial partitions since each of the 13 PCGs was split into the three codon positions. Once estimated best partition scheme and models, the ML tree was built in IQ-TREE with node support values estimated by performing 1000 fast bootstrap replicates but also computing aLRT and aBayes support as implemented in IQ-TREE. Tree topology, model parameter values and node ages were co-estimated and optimized under BI in BEAST. Best partition scheme and nucleotide substitution models were set as determined for ML analysis. Default priors for all parameters were implemented except for clock and tree priors. Three independent analyses were run implementing priors as follows: (i) strict clock and Yule tree priors; (ii) uncorrelated log-normal (UCLN) clock and Yule tree priors; and (iii) UCLN clock and Birth-Death tree priors. We aimed to investigate the phylogenetic relationships between *Palaemon* species with particular attention to the putative cryptic species existing within *P*. *elegans*. For that reason, we calibrated the tree by constraining the age of the divergence between all the *P*. *elegans* samples according to the geological age of the Messinian Salinity Crisis (MSC). The mrca of the three *P*. *elegans* samples was constrained with a log-normal distribution prior in real space with a mean (M) of 5.637 Ma and a standard deviation (SD) of 0.16 (95% confidence interval 5.33-5.96 Ma, [24]). Bayesian analyses were run for 50 million generations, sampling every 5000 generations. Convergence of the runs was assessed in Tracer v1.7 [45] ensuring parameter values reached effective sample size (ESS) above 200 after convergence. Mean values and 95% confidence intervals of parameters and ages of each run were estimated in TreeAnnotator v1.8.4 (BEAST) after a burn-in of the first 5 million generations. Different clocks and tree diversification models were statistically compared based on Bayes Factors (BFs), which were calculated from marginal likelihoods estimated using path-sampling and the stepping-stone model as implemented in BEAST. We performed 100 steps of one million generations each using a path scheme with a betaQuantile 0.33 [46], discarding 25% of the run as burn-in. Patristic distances were estimated with the software Patristic [47] using the ML tree.

Lastly, since nucleotide substitution rates from invertebrate taxa are generally estimated using short *cox1* sequences only instead of full mitochondrial PCGs, we estimated the rate of our *cox1* sequences to compared it with those rates reported in literature. Therefore, another BEAST analysis was run exclusively using *cox1* sequences and implementing the partition scheme, substitution and clock models, and tree priors previously inferred for the 13 concatenated PCGs. Tree topology was also fixed according to the best phylogenetic result for the PCGs in BEAST. As a single gene was used for this analysis, we constrained the age of two nodes in order to calibrate the tree properly: (i) the MSC for *P*. *elegans* as defined above; and (ii) the age of the root as a log-normal distribution in real space with M = 130.4 Ma and a SD = 23 (CI 91.13-181 Ma) as previously estimated in the phylogeny of the concatenated PCGs. Phylogenetic trees were visualised in FigTree v1.4.4 [48].

## Results

### Mitochondrial genomes

The mitogenome of seven out of the 10 sequenced samples could be assembled and annotated (Table 2). The mitogenomes of *P*. *adspersus*, *P*. *elegans* (Atlantic), *P*. *elegans* (Mediterranean), *P*. *serratus* (Atlantic) and *P*. *varians* were *de novo* assembled from high quality reads using SPAdes. On the other hand, the mitogenome sequences of *P*. *elegans* (cryptic species) and *P*. *longirostris* were obtained by iterative mapping of high quality reads on the mitochondrial genome of *P*. *adspersus*. In detail, the mitogenomes of *P*. *adspersus* and *P*. *serratus* (Atlantic) were obtained as complete and circular, whereas some mitochondrial regions could not be filled in the mitogenomes of *P*. *elegans* (Atlantic), *P*. *elegans* (Mediterranean), *P*. *elegans* (cryptic species), *P*. *longirostris* and *P*. *varians* because they exhibited no reads coverage. Extremely low coverage even prevented mitogenome assembly for *P*. *macrodactylus*, *P*. *serratus* (Mediterranean) and *P*. *zariquieyi*, being only possible to retrieve some single gene sequences for those samples. Thus, unevenness of coverage was observed across our assembled mitogenomes (S1 Fig), where mitogenome contigs showed regions with high coverage values adjacent to blocks with low ones in spite of having large mean coverage values. Besides, regions with lower coverages were linked to those having higher G+C percentages, i.e. inverse correlation (S1 Fig).

**Table 2:** Sequence statistics of the *Palaemon* mitochondrial genomes assembled and annotated in this study.

Sample	Length (bp)	A%	C%	G%	T%	%AT	AT skew	GC skew
***Palaemon adspersus***	15736	0.344	0.202	0.130	0.324	66.8	0.030	-0.215
***Palaemon elegans* (Atlantic)**	15700	0.311	0.216	0.154	0.312	62.4	-0.002	-0.170
***Palaemon elegans* (Mediterranean)**	15834	0.309	0.218	0.156	0.313	62.2	-0.006	-0.168
***Palaemon elegans* (cryptic species)**	15748	0.278	0.190	0.132	0.274	55.2	0.006	-0.180
***Palaemon longirostris***	15733	0.323	0.194	0.124	0.306	62.9	0.028	-0.220
***Palaemon serratus* (Atlantic)**	15758	0.340	0.200	0.133	0.328	66.8	0.018	-0.201
***Palaemon varians***	14889	0.308	0.225	0.159	0.308	61.6	-0.001	-0.170

**Fig 2:**
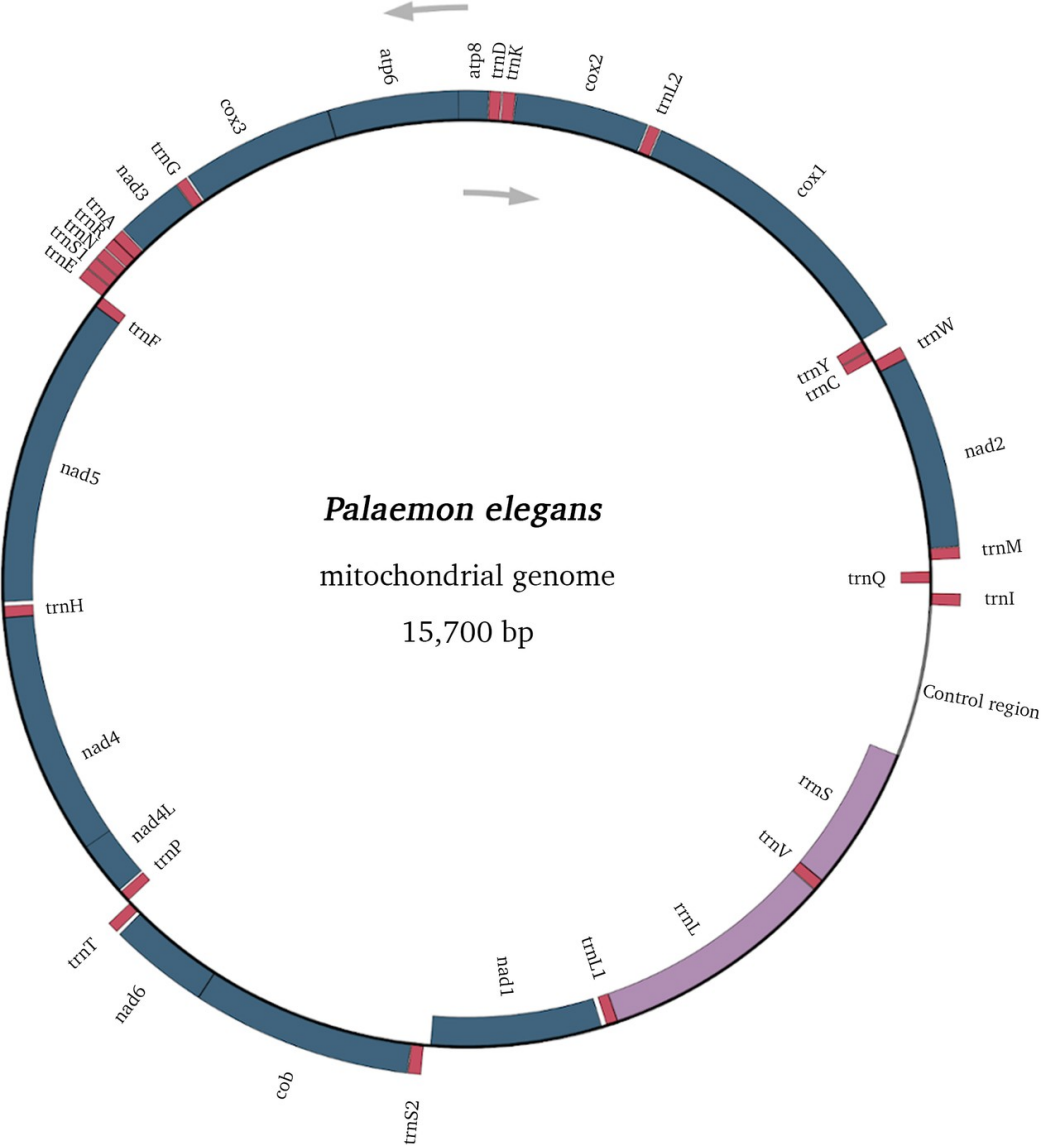
Mitochondrial genome map of *Palaemon elegans*. Genes are shown using standard nomenclature. Genes outside the map are encoded on + strand and transcribed in clockwise direction, whereas genes inside the map are encoded on - strand and transcribed in counterclockwise direction.

Mitogenome sequence statistics for these seven *Palaemon* samples are summarised in Table 2. Most length differences among samples were attributed to regions that could not be fully recovered due to lack of coverage, especially the control region. Complete mitogenome was achieved for *P*. *adspersus* and *P*. *serratus* and they were 15,736 bp and 15,758 bp in length, respectively. A+T content varied from 55.2% for *P*. *elegans* (cryptic species) to 66.8% for *P*. *adspersus* and *P*. *serratus*. As for intrastrand skewness, all mitogenomes exhibited an almost balanced composition for A and T, ranging AT skew ([A-T]/[A+T]) from -0.006 (*P*. *elegans* Mediterranean) to 0.030 (*P*. *adspersus*). On the contrary, all mitogenomes showed negative GC skew ([G-C]/[G+C]) ≤ -0.168, indicating a C bias. Mitochondrial genomes in *Palaemon* encoded the 37 genes characteristic of metazoan mitogenomes, including 13 PCGs, 2 rRNA genes and 22 tRNA genes, and one control region (Fig 2 and S1 Table). Same gene arrangement was found in all analysed *Palaemon* samples. Twenty-three genes were encoded on the + (heavy) strand, while the remaining 14 genes were encoded on the - (light) strand. Intergenic nucleotides and overlapping gene sequences were found in all the studied mitogenomes. Genes containing unknown nucleotides (Ns) and genes that could not be annotated because their sequence was missing or partially incomplete are specified per sample in S1 Table.

Regarding PCGs, nine of them were located on the + strand (*atp6*, *atp8*, *cob*, *cox1*, *cox2*, *cox3*, *nad2*, *nad3* and *nad6*), while four genes were on the - strand (*nad1*, *nad4*, *nad4L* and *nad5*; Fig 2). Nucleotide composition and skewness of PCGs over samples are plotted on S2 Fig. A+T content varied within codons in both strands, being the highest in third codon position (average 69.2%), followed by second (60.7%) and then first position (57.6%). Across PCGs, AT% ranged from 59.5% (*cox3*) to 67% (*atp8*). PCGs showed a general bias towards T (average AT skew = -0.178), being the highest occurrence of T compared to A in second codon positions (AT skew = -0.443) and in the *nad4L* gene (-0.320). The variation in intrastrand GC skew was greater, although GC skew averaged -0.062, indicating fairly balanced occurrence of G and C. Strong G bias was observed in the PCGs encoded by the - strand (highest GC skew in *nad4L*), while the PCGs encoded by the + strand showed strong C bias (lowest GC skew in *atp8*). Most PCGs were initiated by standard ATN codons, but uncommon start codons ACG, GTG and TTA were also found (S1 Table). As in the previously analysed *Palaemon* mitogenomes, *cox1* used ACG as initiation codon in our species. *Palaemon elegans* samples used GTG as start codon in *nad4*, and *nad5* employed TTA as start codon only in *P*. *varians*. Most PCGs terminated with canonical TAA and TAG as stop codons while others terminated as truncated T (S1 Table). Leu (16.30%), Ser (9.60%), Phe (7.80%) and Ile (7.30%) were the most used amino acids across PCGs, meanwhile Cys (1.20%), Arg (1.60%), Asp (1.90%) and Gln (2%) were the least frequently coded.

Both rRNA genes were located on the - strand, and similarly to the PCGs encoded by this strand, they were skewed in favour of G (GC skew = 0.222). *rrnL* (16S) and *rrnS* (12S) genes were located between *trnL1* and *trnV* genes, and between *trnV* gene and control region, respectively. The complete set of twenty-two tRNAs were identified in *Palaemon* mitogenomes, with two leucine and two serine tRNA genes differentiated by their anticodon sequences. Sequence length of tRNA genes ranged from 62 (*trnA*) to 70 (*trnC* and *trnS2*) bp in size. Fourteen tRNAs were located in the + strand, whilst the remaining eight genes were on the - strand. tRNAs displayed the typical cloverleaf secondary structure, except *trnS1* whose DHU arm has been lost as in most metazoans (S3 Fig). Mismatches were detected in a few base pairs within tRNAs and some of them were fully conserved across our *Palaemon* samples, namely in *trnC*, *trnE*, *trnK*, *trnL1*, *trnR* and *trnW*. A wobble mismatch was observed in *trnV* in all samples excepting *P*. *varians*. The non-coding control region only could be completely recovered for *P*. *adspersus* and *P*. *serratus*, being located between *rrnS* and *trnI* genes, and 946 and 976 bp in length, respectively. In both species, the control region was the mitogenomic region with the highest A+T content, 73.3% in *P*. *serratus* and 79.2% in *P*. *adspersus*.

### Phylogenetic analyses

Phylogenetic analyses were based on the nucleotide sequences of the 13 mitochondrial PCGs from the ten *Palaemon* samples analysed in this study, plus other seven *Palaemon* species and four outgroups belonging to the genus *Macrobrachium* (Table 3). The individual alignments of the 13 PCGs were concatenated in a single dataset of 11,097 bp (S1 File). As mentioned above, the mitochondrial genome of 3 out of the 10 *Palaemon* samples sequenced could not be assembled because some regions lacked coverage. Consequently, all PCGs could be recovered for *P*. *macrodactylus* but with some Ns, while only a few single PCG fragments could be recovered for *P*. *serratus* (Mediterranean) (*atp6*, *cob* and *nad5*) and *P*. *zariquieyi* (*atp6*, *cob*, *cox1*, *cox2*, *cox3*, *nad4* and *nad5*). According to Bayesian Information Criterion (BIC) scores, the best partition scheme consisted of subdividing the nucleotide dataset into five partitions: 1st codon positions encoded on the + strand; 1st codon positions encoded on the - strand (genes *nad1*, *nad4*, *nad4L* and *nad5*); 2nd codon positions; 3rd codon positions encoded on the + strand; and 3rd codon positions encoded on the - strand. Best nucleotide substitution model for each partition resulted in GTR+F+I+G4, excepting the 1st codon positions encoded on the - strand for which the best model was TN+F+I+G4.

**Table 3:** List of the species whose mitochondrial genome sequences were used for phylogenetic analyses.

Species	Accession No	Sequence	Reference
*P*. *adspersus*	MT340092	Mitogenome	This study
*P*. *elegans* (Atlantic)	MT340089	Mitogenome	This study
*P*. *elegans* (Mediterranean)	MT340087	Mitogenome	This study
*P*. *elegans* (cryptic species)	MT340088	Mitogenome	This study
*P*. *longirostris*	MT340091	Mitogenome	This study
*P*. *serratus* (Atlantic)	MT340086	Mitogenome	This study
*P*. *varians*	MT340090	Mitogenome	This study
*P*. *macrodactylus*	MT340174-86	Single genes	This study
*P*. *serratus* (Mediterranean)	MT340187-89	Single genes	This study
*P*. *zariquieyi*	MT340190-96	Single genes	This study
*P*. *annandalei*	NC_038117	Mitogenome	Yuan *et al*. [49]
*P*. *capensis*	NC_039373	Mitogenome	Wood *et al*. [50]
*P*. *carinicauda*	NC_012566	Mitogenome	Shen *et al*. [51]
*P*. *gravieri*	NC_029240	Mitogenome	Kim *et al*. [52]
*P*. *modestus*	MF687349	Mitogenome	Wang *et al*. [53]
*P*. *serenus*	NC_027601	Mitogenome	Gan *et al*. [54]
*P*. *sinensis*	MH880828	Mitogenome	Zhao *et al*. [55]
**Outgroups:**			
*M*. *rosenbergii*	NC_006880	Mitogenome	Miller *et al*. [56]
*M*. *lanchesteri*	NC_012217	Mitogenome	Unpublished
*M*. *bullatum*	NC_027602	Mitogenome	Unpublished
*M*. *nipponense*	NC_015073	Mitogenome	Ma *et al*. [57]

Our ML phylogenetic tree (S4 Fig) showed maximum support (bootstrap value = 100, and aBayes and aLRT = 1) in almost all nodes and retrieved two main palaemonid mitogenome lineages, one comprised all the *Macrobrachium* species included as outgroups along with *P*. *capensis*, whereas the second lineage grouped the rest of the species of the genus *Palaemon*. Hence, the genus *Palaemon* did not conform monophyla as in previous studies. Within the *Palaemon* lineage, *P*. *sinensis* occupied a basal position while the other species were split into two clades. One clade was formed by the Asia-Pacific species (except *P*. *serenus*), with *P*. *macrodactylus* and *P*. *gravieri* being the sister species of the group composed by the species that belonged to the synonymised genus *Exopalaemon* (*P*. *annandalei*, *P*. *carinicauda* and *P*. *modestus*). The second *Palaemon* clade consisted in all the European species plus the Australian species *P*. *serenus*, which was the most basal and the only Asia-Pacific species placed in this clade. European distributed species were further subdivided into two smaller clades, one comprising species that belonged to the synonymised genus *Palaemonetes* (*Palaemon varians* and *P*. *zariquieyi*) and the other including the remaining species (*P*. *elegans*, *P*. *longirostris*, *P*. *adspersus* and *P*. *serratus*). The cryptic species found within *P*. *elegans* was indeed revealed as the sister species of the Atlantic and Mediterranean populations of *P*. *elegans*. Pairwise patristic distance matrix was calculated from the ML tree (S2 Table). Patristic distance between the cryptic species of *P*. *elegans* and the Atlantic and Mediterranean populations of *P*. *elegans* was almost identical, 0.150 and 0.148, respectively. As for the phylogenetic distance between populations of the same species, it was highly uneven, being 1.7% for *P*. *elegans* and 25.2% for *P*. *serratus*.

**Fig 3:**
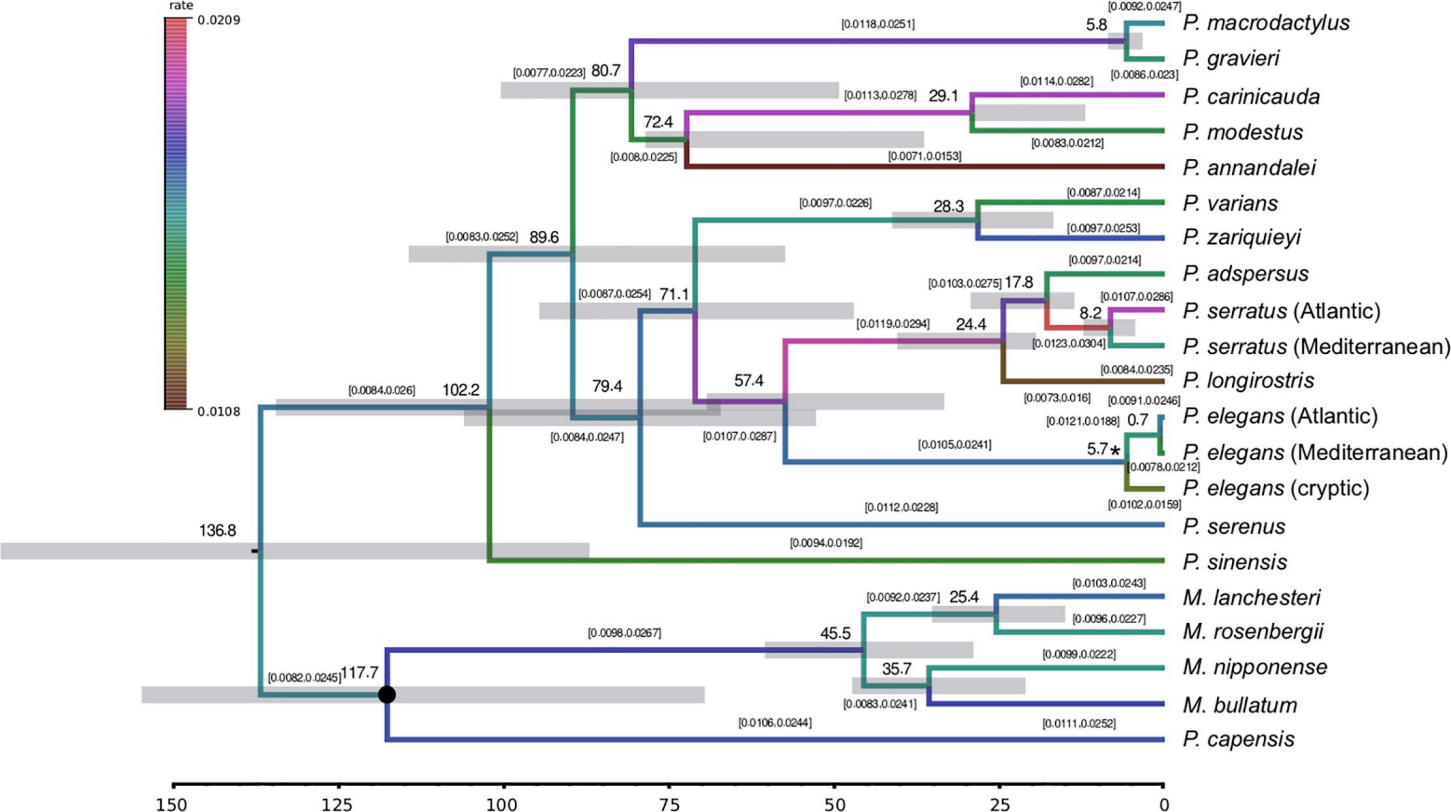
Calibrated tree showing divergence times for *Palaemon* and *Macrobrachium* species estimated from the Bayesian analysis of the 13 mitochondrial protein-coding genes implementing the best selected partition, nucleotide substitution and clock models. Bars across nodes represent the 95% highest probability density intervals for the estimation of node ages. The Messinian Salinity Crisis was used as aged constraint and is indicated with an asterisk. Time in million years before present. Brackets indicate the 95% highest probability density intervals for the substitution rate estimation for each branch.

Regarding calibration analysis, the best fitting clock model resulted from setting one independent uncorrelated log-normal relaxed (UCLN) clock and a Yule diversification model (Table 4). Birth-Death diversification model could not be statistically discarded by Bayes Factors (BF within range from 1 to 3.2) but it was worse than Yule model and it involves more parameters, so it was not used for further analyses. The BI phylogenetic tree displayed the same topology as the ML tree, showing maximum support (posterior probability = 1) in almost all nodes. The MSC (5.33-5.96 Ma, late Miocene) was the geological event used as node constraint to calibrate de Bayesian tree (Fig 3), as it was postulated as the most likely scenario that gave rise to the cryptic species found within *P*. *elegans*. Thus, the divergence between the cryptic species and *P*. *elegans* was forced to dated back at 5.3-5.9 Ma, matching with the MSC. The root of the tree was estimated to fall in the Lower Cretaceous, about 136.8 Ma (95% highest posterior density [HPD95%] = 87.1-176.2 Ma). According to our analysis, the differentiation of *P*. *elegans* across the Atlantic-Mediterranean transition area started circa 0.7 Ma (HPD95% = 0.3-0.9 Ma) in the Pleistocene, so it might be related to the recurrent glaciation episodes that took place at that time. Conversely, the separation of the Atlantic and Mediterranean populations of *P*. *serratus* was estimated to be much older, at about 4.5-12.3 Ma (mean value 8.2 Ma) in the Plio-Miocene. Despite the Mediterranean sample of *P*. *serratus* showed a congruent phylogenetic position, their estimates should be carefully evaluated since its concatenated sequence had a high amount of missing data.

**Table 4:** List of Bayesian analyses constrained under different molecular clocks and diversification models ranked by Bayes Factors.

Scheme and models	Path-sampling		Stepping-stone	
	Marginal Ln	Bayes Factors	Marginal Ln	Bayes Factors
**5 partitions 1 strict Yule**	-107348.769	85.735	-107348.291	86.328
**5 partitions 1 UCLN Yule**	-107305.902	0.000	-107305.127	0.000
**5 partitions 1 UCLN Birth-Death**	-107306.636	1.469	-107306.045	1.836

Marginal likelihoods were estimated using both path-sampling and stepping stone methods, whose values were used to calculate Bayes Factors. Dataset was partitioned as five partitions (1st codon plus strand, 1st codon minus strand, 2nd codon, 3rd codon plus strand, 3rd codon minus strand) according to BIC score (215,696.611). Abbreviations: UCLN (uncorrelated log-normal clock) and strict (strict clock).

The mean nucleotide substitution rate estimated for the combined 13 mitochondrial PCGs was 1.59×10^−2^ substitutions per site, million years and lineage, with a 95% confidence interval (HPD95%) 1.09-2.15×10^−2^. This value corresponds to an absolute value of 1.59×10^−8^ substitutions per site, year and lineage, and a pairwise divergence of 3.18% per million years. In order to check the impact of the missing data of *P*. *macrodactylus*, *P*. *serratus* and *P*. *zariquieyi* on the calibration results, we performed another BEAST analysis removing these species from the dataset. The same tree topology was retrieved and only some slight changes were observed in node ages and in the mean mitogenome substitution rate (1.57×10^−2^ per site, million years and lineage). However, as mitochondrial substitution rates for decapods have been primarily based on *cox1*, in addition to providing the first substitution rate for palaemonid mitogenomes, we also estimated the substitution rate for *cox1*. The mean substitution rate estimated for *cox1* was 1.41×10^−2^ per site, million years and lineage (HPD95% = 0.99×10−2-1.86×10^−2^), what equals to a 2.82% of divergence.

## Discussion

This work describes the mitochondrial genome of six *Palaemon* species for the first time, being one of them the cryptic species previously reported within *P*. *elegans* [12,13]. They are also the first European species of the genus *Palaemon* to have their mitogenome characterized. Moreover, the mitogenomes of both Atlantic and Mediterranean lineages of *P*. *elegans* were obtained, so this study largely increase the available sequence resources for the genus *Palaemon*.

Although samples of *P*. *macrodactylus*, *P*. *serratus* (Mediterranean) and *P*. *zariquieyi* were also sequenced, their mitogenomes could not be assembled due to many mitochondrial regions lacked coverage, so only gene fragments could be recovered. We invoked failures during library preparation or the usage of Nextera XT DNA Library Prep kit to explain that lack of coverage. Severe drops in coverage across mitogenomes have been observed when using Nextera XT for library preparation [58–61]. Low-coverage regions have been attributed to bias in the Nextera XT transposase reaction and/or in the library amplification of GC-rich regions [61]. As great variability in coverage was consistent across our samples and coverage showed an inverse correlation relative to GC content, we considered that Nextera XT was the most likely source.

Sequence length and base composition of the seven mitogenomes described here were in line with those previously reported for other *Palaemon* mitogenomes. Similarly, gene content and order of our mitogenomes were also the same as for the *Palaemon* species with known mitogenome, sharing all of them an inversion between *trnP* and *trnT* genes that deviates from the primitive pancrustacean mitogenome order [62]. This reversal seems to be a unique mitochondrial feature of the genus *Palaemon*, as it was not detected in the related genus *Macrobrachium*. Gene rearrangements occur commonly in Malacostraca mitochondrial genomes [51,63–65] and could be useful for understanding the mitogenome evolution within this group.

Phylogenetic analyses were conducted based on the concatenated mitochondrial PCG sequences of our ten *Palaemon* samples, plus sequences from other seven *Palaemon* species taken from GenBank and four *Macrobrachium* species used to root the trees. As a result, here we provide the largest mitogenome phylogeny for the genus *Palaemon* to date, where all nodes were strongly supported. Although extensive worldwide phylogenetic analyses have been performed in *Palaemon*, they were always based on a few mitochondrial (*cox1* and 16S rDNA) and/or nuclear (histone 3 and 18S rDNA) genes [7–10]. In this regard, our trees showed a general agreement with the last phylogenetic assessment of the genus [8], supporting the current non-monophyletic status of *Palaemon* since the South African species *P*. *capensis* was included in the lineage comprising the *Macrobrachium* species. *Palaemon capensis* was also placed in the *Macrobrachium* clade in a previous mitogenome phylogeny [55]. The genus *Macrobrachium* comprises over 200 freshwater prawns globally distributed across tropical and subtropical regions [66]. Carvalho *et al*. [8] already reported that *Palaemon* species with freshwater phases exhibited a closer relationship with the genus *Macrobrachium* than to other *Palaemon* species, and were included in one of the three clades identified within *Palaemon*, the ‘*Alaocaris’* clade. Consequently, *P*. *capensis* may be included in the *‘Alaocaris’* clade as is an amphidromous shrimp [67]. In any case, our phylogenetic analyses highlighted that future systematic changes are still required in *Palaemon* and, more specifically, that *P*. *capensis* should be excluded from this genus. With the exception of *P*. *capensis*, all *Palaemon* species included in our analyses belong to the *‘Palaemon sensu stricto’* clade defined by Carvalho *et al*. [8]. Species belonging to the no longer valid genera *Exopalaemon* (*P*. *annandalei*, *P*. *carinicauda* and *P*. *modestus)* and *Palaemonetes (Palaemon sinensis*, *Palaemon varians* and *P*. *zariquieyi*) were included in our dataset and their phylogenetic positions proved that they were properly synonymised with *Palaemon* [2]. Species of the *Palaemon* lineage were divided into two main clades according to their geographical distribution, indicating that the current distribution pattern of *Palaemon* is better explained in terms of vicariance rather than of dispersal. Species distributed throughout the Asia-Pacific region were grouped together in one clade, while European distributed species were joined in the other clade along with the endemic Australian species *P*. *serenus*. Notwithstanding, in previous phylogenies based on single genes [8–10,68], *P*. *serenus* was more related with Asia-Pacific species than with European species. Further analyses based on an intensive sampling effort and mitogenome phylogenetics to reconstruct the ancestral state of the geographical distribution of the genus are needed to fully understand the diversification pattern of *Palaemon* species across all continents.

We particularly aimed to shed light on the cryptic species previously found within *P*. *elegans*. Marine habitats are fertile grounds for crypticism as phenotypic plasticity of marine invertebrates, such as *Palaemon* shrimps, hinders the discrimination of closely related species by traditional morphological analysis [69]. Both mitochondrial [12] and microsatellite [13] markers revealed clear evidence for the existence of a Mediterranean cryptic sympatric species that was unambiguously identified as *P*. *elegans* by morphology. Here the cryptic species was verified as the sister species of the Atlantic and Mediterranean populations of *P*. *elegans*. In fact, phylogenetic distance between the cryptic species and *P*. *elegans* account for a 15%, much more than expected between populations of the same species and comparable to the degree of divergence between congeneric species. This distance reflected a degree of divergence between the cryptic species and *P*. *elegans* similar to that found between *P*. *macrodactylus* and *P*. *gravieri* (18.1%), formally accepted as different species. Therefore, crypticism within *P*. *elegans* was disclosed once again and confirmed in a phylogenetic frame.

The native distribution of *P*. *elegans* ranges from the eastern Atlantic Ocean (from Scotland and Norway to Mauritania, including the Azores, Madeira and Canary Islands) to the entire Mediterranean Sea and the Black Sea [70]. However, the cryptic species of *P*. *elegans* was exclusively recorded in the Mediterranean Sea, so its origin should be located on that basin. The Mediterranean Sea has a tortuous geological history that largely contributed to its high level of biodiversity and endemism [71]. Specifically, it has been postulated that the MSC in late Miocene, [24] was the most likely scenario that gave rise of this cryptic species [12,13]. To further test this hypothesis, we used the MSC as calibration point for the mrca of *P*. *elegans* to estimate mitochondrial PCGs substitution rate in *Palaemon* and compare results to those attained in other pancrustacean species. We provided the first time-calibrated phylogenetic tree and the first molecular clock for the full set of mitochondrial PCGs (1.59% per million years) in Decapoda. This rate is within the range of the few mitochondrial genome substitution rates estimated for other arthropods [20,72,73]. Furthermore, since most substitution rates have been based on *cox1* only, we also estimated its rate (1.41% per million years) and it is effectively in the range of those rates reported in literature for this gene in decapods [7,11,74–76]. As both mitochondrial PCGs and *cox1* substitution rates described in *Palaemon*, and also in *P*. *elegans*, were compatible with those published elsewhere we concluded that MSC could be a fully plausible cause for the split between *P*. *elegans* and its cryptic species. During the MSC the Strait of Gibraltar was closed by tectonic uplift, isolating the Mediterranean Sea from the Atlantic Ocean and determining the extensive desiccation of the Mediterranean basin [24]. The ancestors of the cryptic species should survived in local refugia in the Mediterranean Sea during the MSC and diverged from Atlantic *P*. *elegans*. With the re-opening of the Strait of Gibraltar, the Mediterranean basin was rapidly flooded again with Atlantic water [77], so Atlantic *P*. *elegans* individuals were re-introduced, but they should no longer be able to reproduce with the cryptic species ancestors, since none genetic hybrids are currently detected [13]. The MSC was also considered the origin of other marine species, as crabs of the genera *Carcinus* [78] and *Maja* [79] or brittle stars of the genus *Ophiothrix* [69]. Thereby, in the present work we corroborated the existence of a cryptic species hidden within *P*. *elegans* and accepted the MSC as the event for its origin. It is essential to thoroughly examine whether there is any potential diagnostic character to discriminate the cryptic species and *P*. *elegans sensu stricto*, as well as to update the systematic consideration of *P*. *elegans*.

According to our calibration analysis, the Atlantic and the Mediterranean populations of *P*. *elegans sensu stricto* differentiated circa 0.3-0.9 Ma, most probably related to the recurrent glaciations of the Pleistocene. Cyclic Pleistocene glaciations involved climatic and sea level regressions that interrupted the connectivity between Atlantic and Mediterranean waters, leaving a footprint on the genetic structure of marine populations [80,81]. In other words, Pleistocene glaciations shaped the genetic diversity of several marine species [e.g. 82–84], and this would be also the case of *P*. *elegans sensu stricto*. Molecular markers have detected population structuring in *P*. *elegans sensu stricto* across the Atlantic-Mediterranean transition area [12,13]. The genetic break that separates these two populations matched with the location of the Almería-Orán Front, a semi-permanent dynamic oceanographic front consisted in two anticyclonic gyres that entails abrupt changes of temperature and salinity [85]. The Almería-Orán Front has been considered as the most important hydrographic boundary to gene flow between the Atlantic and Mediterranean surface waters, acting on the genetic structure of a wide variety of taxa (see review in [86]). Thus, Pleistocene glacial periods would led to genetic differentiation between the Atlantic and Mediterranean populations of *P*. *elegans sensu stricto*, and it is currently maintained by the Almería-Orán Front. The population structure of *P*. *elegans sensu stricto* is therefore the result of past vicariant events and a present-day oceanographic barrier to gene flow.

On the contrary, the estimated age for the divergence between the Atlantic and Mediterranean populations of *P*. *serratus* was far much older (4.5-12.3 Ma, Plio-Miocene), and their phylogenetic distance (25.2%) was considerably greater than expected for populations of the same species, even greater than between *P*. *elegans* and its cryptic species. Overall, our results indicated that the populations of *P*. *serratus* could represent two cryptic allopatric species. The geographical distribution of the common littoral shrimp *P*. *serratus* ranges from the Atlantic Ocean (from Scotland and Denmark to Mauritania, including Azores, Madeira and Canary Islands) to the entire Mediterranean Sea and the Black Sea [70]. Although the estimates involving the Mediterranean sample of *P*. *serratus* must be taken carefully, as only three PCG fragments could be recovered, previous population genetics analyses would support the consideration of these two populations as separated species. Strong genetic differentiation between Atlantic and Mediterranean localities was detected along the geographical distribution range of *P*. *serratus* using two mitochondrial and one nuclear PCG [87] and 17 microsatellite markers [88]. Two geographic lineages were identified in *P*. *serratus* with an unusual phylogeographical break located west of the Strait of Gibraltar in the Gulf of Cádiz. In the light of those findings, these two lineages were attributed to be different populations of *P*. *serratus*, as no consistent morphological differences were found among them [88]. Here we presumed that Atlantic and Mediterranean lineages could be actually two allopatric species rather than populations of the same species. The MSC is younger than the divergence between the populations of *P*. *serratus* (mean value 8.2 Ma) so even though it does not appear to be the primary cause for their split, it could not be completely ruled out since it falls within the HPD95% of that age estimate (4.5-12.3 Ma). However, before the onset of the MSC, the connection between the Atlantic Ocean and the Mediterranean Sea was progressively restricted in the late Tortonian (Miocene) by tectonic uplifts and closure of seaways in southern Spain and northern Morocco [89], being the most famous consequence the Tortonian Salinity Crisis (TSC) of the eastern Betics at 7.6 Ma [90]. These changes recorded in the different corridors (7-8.3 Ma) induced disruptions in the water circulation between the Atlantic Ocean and the Mediterranean Sea and sea-level variations [91]. Hence, we preferably consider that the divergence between the Atlantic and Mediterranean populations of *P*. *serratus* could started by restricting their gene flow in the late Tortonian in response to the impact of the cessation of pre-MSC gateways. Subsequent repeated isolations between the Atlantic Ocean and the Mediterranean Sea during the MSC and Pleistocene glaciations could have assisted to maintain enough divergence allowing cryptic allopatric speciation. Further species delimitation studies focused on *P*. *serratus* should be carried out to determine with certainty if it is a cryptic species complex, and subsequently to accurately date the age of that speciation.

In conclusion, we provide the largest mitogenome phylogeny of *Palaemon*, demonstrating that systematic changes are still needed in this genus to conform monophyly. The full mitochondrial genome molecular clock was estimated for the first time in Decapoda by using the MSC for tree calibration. The existence of a cryptic species within *P*. *elegans* was confirmed and the MSC was accepted as the event for its origin. Likewise, we also pointed out that the populations of *P*. *serratus* could also represent two separated species, so this work sheds light on species crypticism in the genus *Palaemon*.

## Supporting information

S1 Fig (a) Coverage across mitogenome sequence of *P*. *adspersus*. (b) Coverage vs percentage of G+C content in log scale across mitogenome sequence of *P*. *adspersus*. (c) Coverage across mitogenome sequence of *P*. *elegans* (cryptic species). Coverage and G+C percentages were estimated using a window size of 100 bp and steps of 50 bp.
(SVG)

S2 Fig **A+T content boxplot for (a) PCGs and for (b) PCGs, tRNAs and rRNAs by codon position and strand. AT skew boxplot for (c) PCGs and for (d) PCGs, tRNAs and rRNAs by codon position and strand. GC skew boxplot for (e) PCGs and for (f) PCGs, tRNAs and rRNAs by codon position and strand.** Statistics were calculated considering the seven *Palaemon* mitochondrial genomes assembled and annotated in this study.
(PDF)

S3 Fig Secondary structures of the 22 tRNA genes encoded by the mitochondrial genome of *Palemon elegans*. tRNAs are referred by single letter amino acid codes.
(PDF)

S4 Fig Maximum Likelihood tree based on the nucleotide sequences of the 13 mitochondrial protein coding genes implementing the selected partitioning scheme. Numbers on nodes indicate support values for aBayes/aLRT/bootstrap.
(PDF)

S1 Table Features of genes encoded in mitochondrial genomes. Asterisk indicates genes containing unknown nucleotides (Ns). Unannotated indicates not annotated genes due to lack of coverage.
(XLSX)

S2 Table Pairwise patristic distance matrix based on the maximum likelihood phylogenetic tree. (XLSX)

S1 File Alignment of 13 concatenated mitochondrial PCGs used for phylogenetic analyses. (TXT)
