# Supplementary figures and images for: Mitogenome phylogenetics in the genus Palaemon (Crustacea: Decapoda) sheds light on species crypticism in the rockpool shrimp P. elegans
Source: PLoS One. 2020 Aug 18;15(8):e0237037. doi: 10.1371/journal.pone.0237037 (PMC7444591; doi:10.1371/journal.pone.0237037)

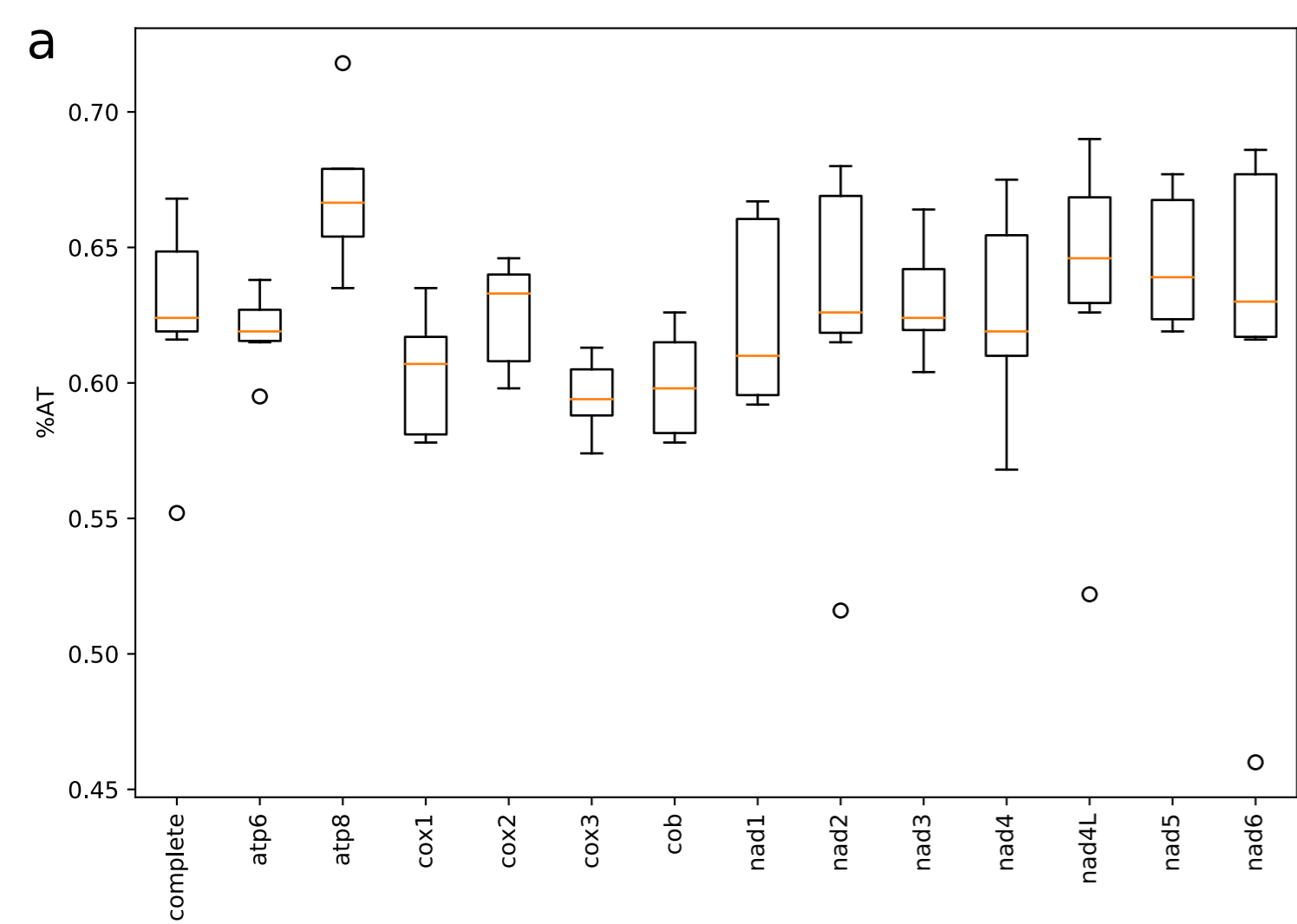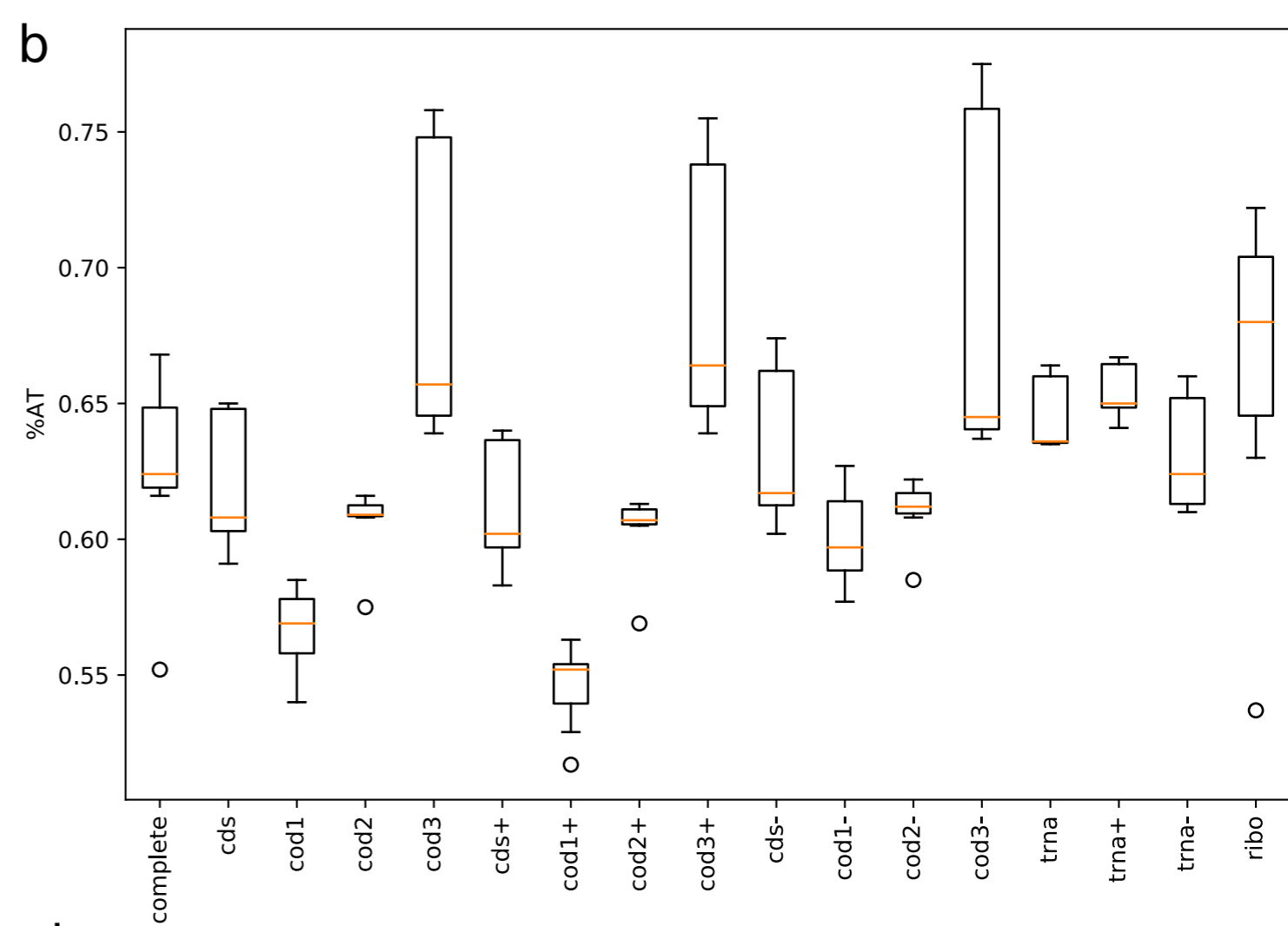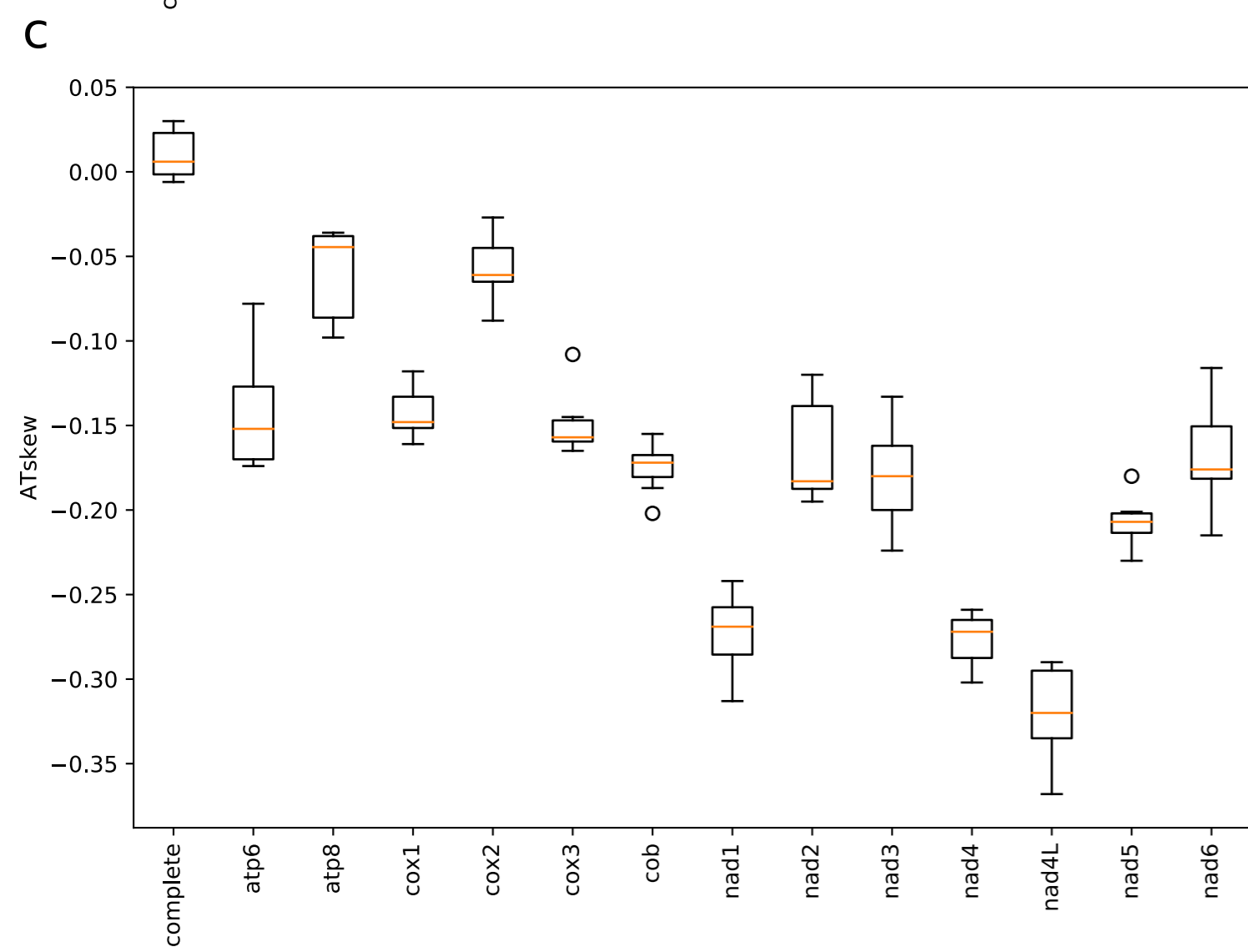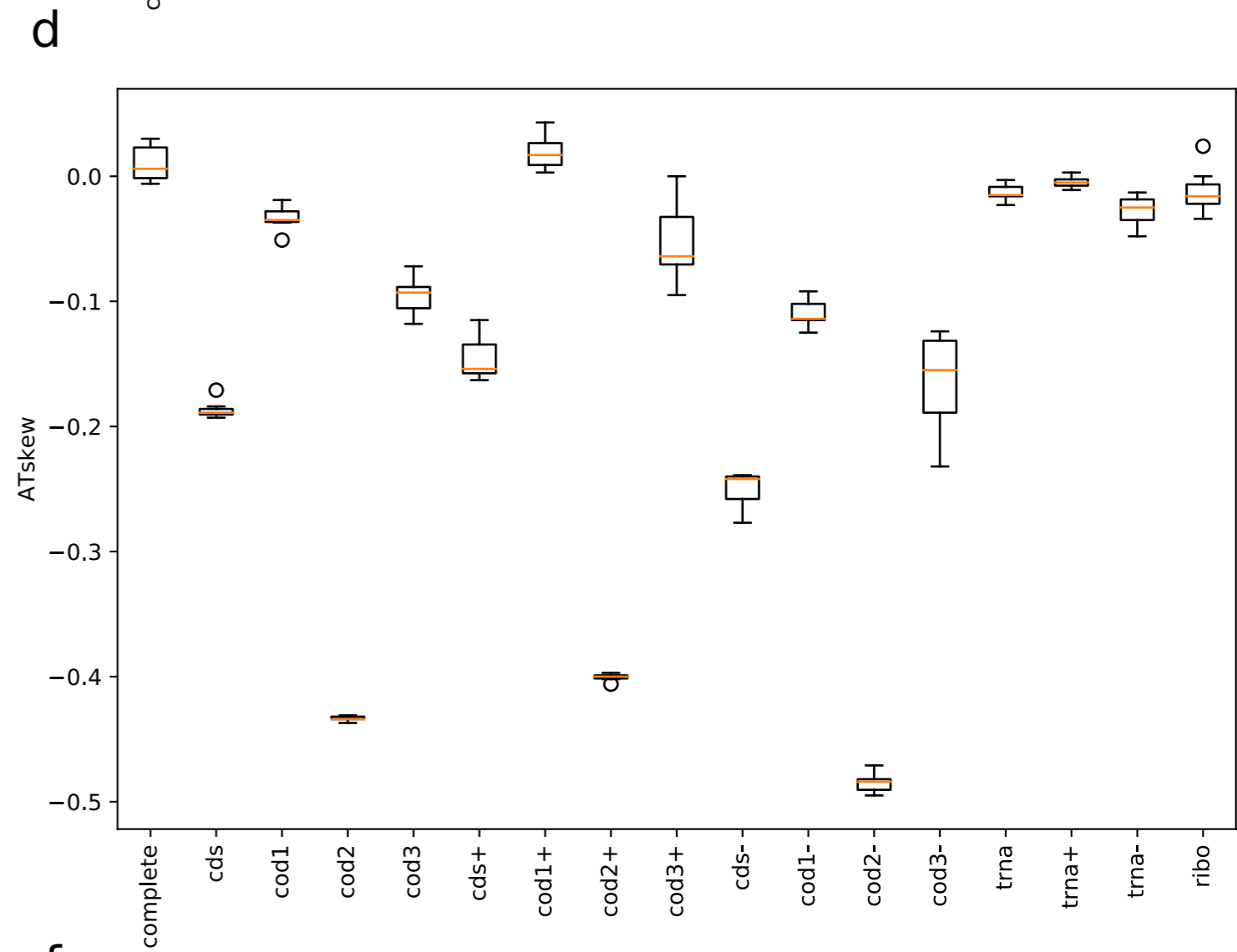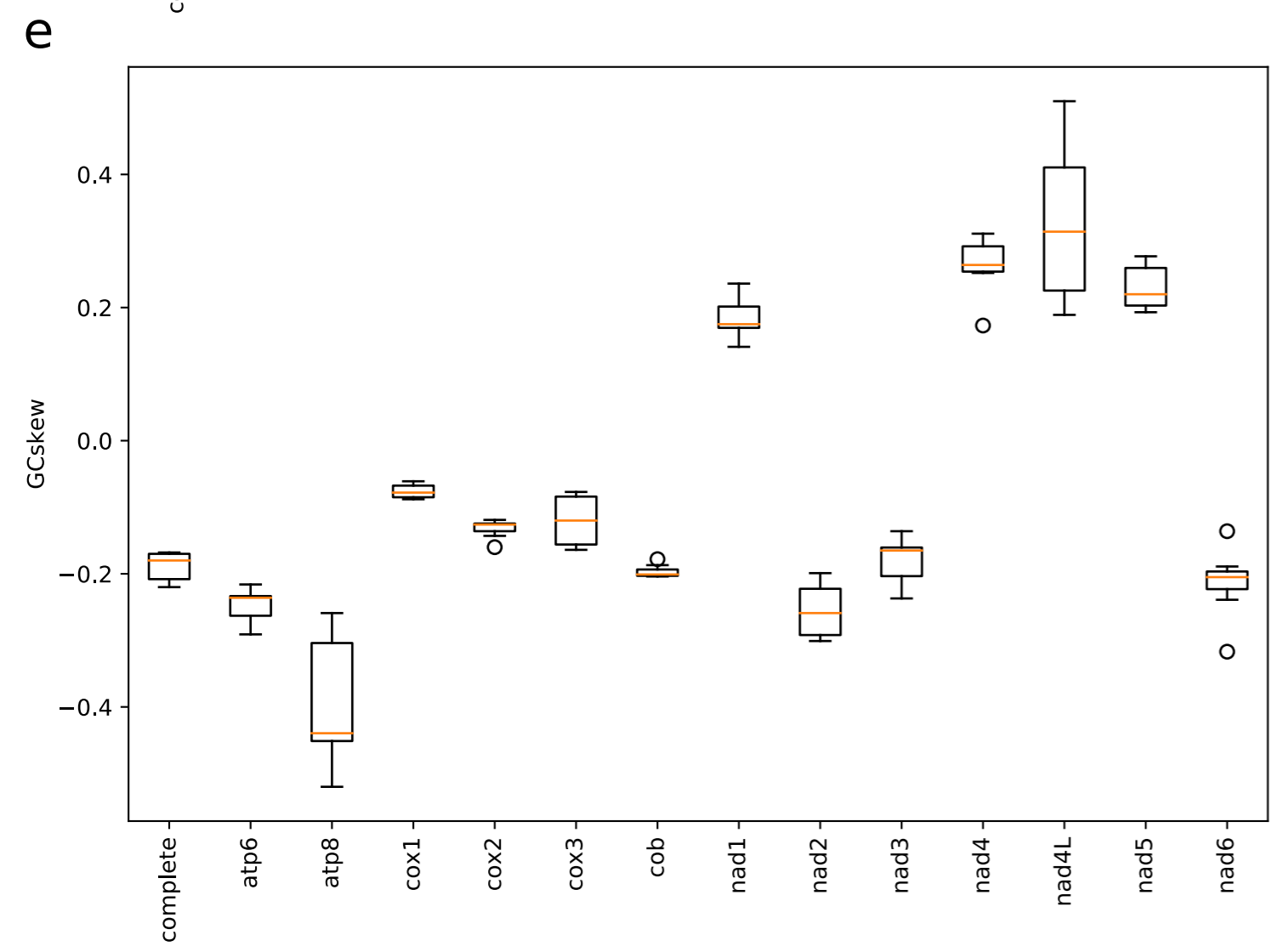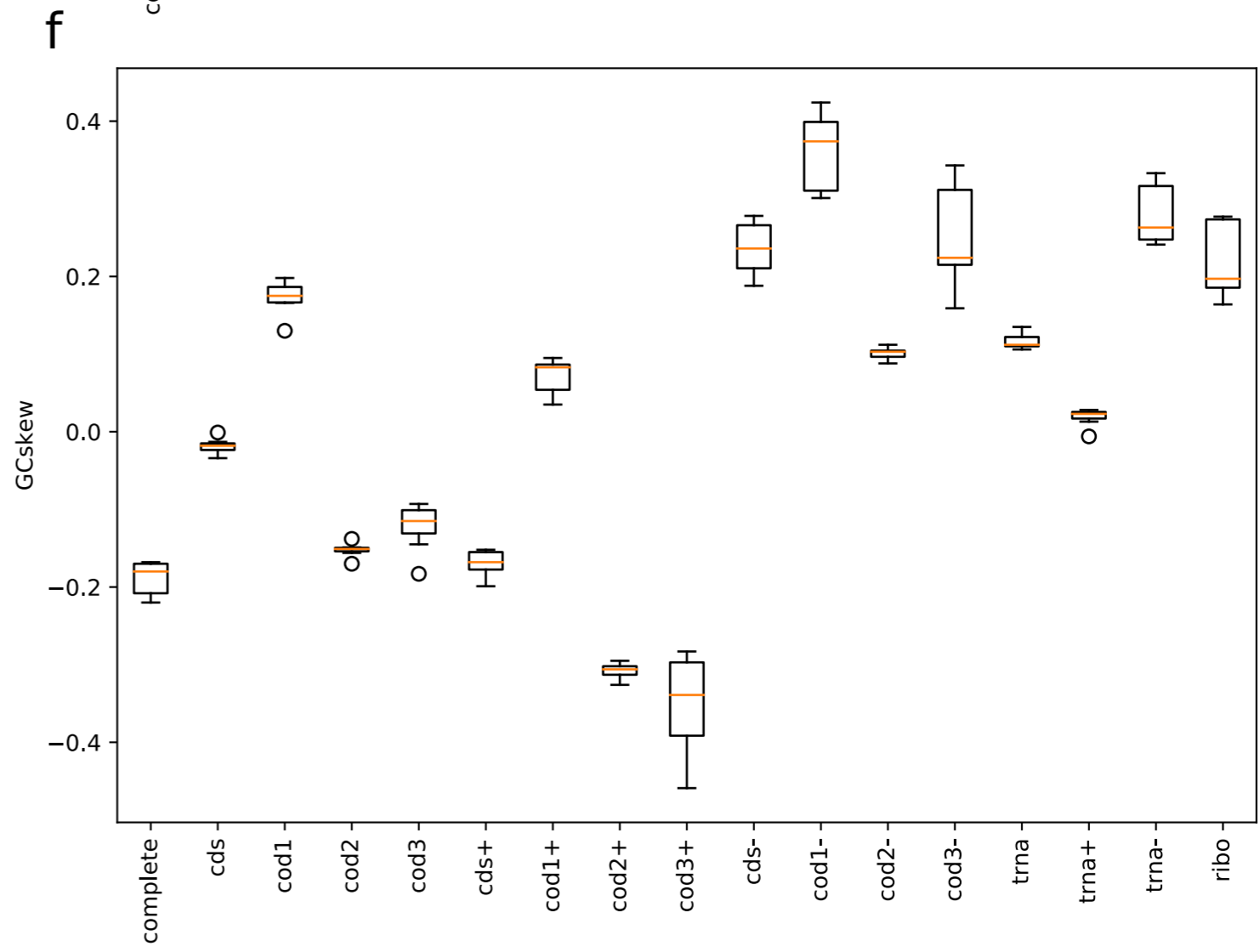

Supplement: S2 Fig — A+T content boxplot for (a) PCGs and for (b) PCGs, tRNAs and rRNAs by codon position and strand. AT skew boxplot for (c) PCGs and for (d) PCGs, tRNAs and rRNAs by codon position and strand. GC skew boxplot for (e) PCGs and for (f) PCGs, tRNAs and rRNAs by codon position and strand. Statistics were calculated considering the seven Palaemon mitochondrial genomes assembled and annotated in this study. (PDF) [file pone.0237037.s002.pdf]

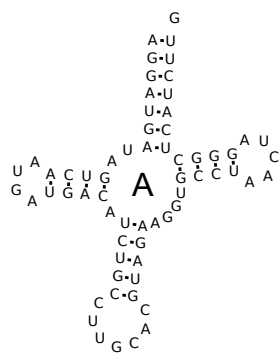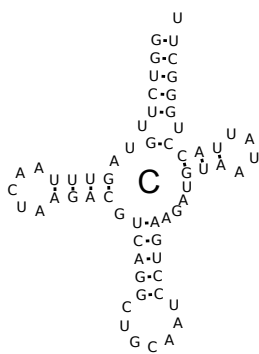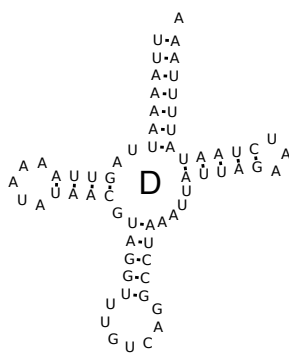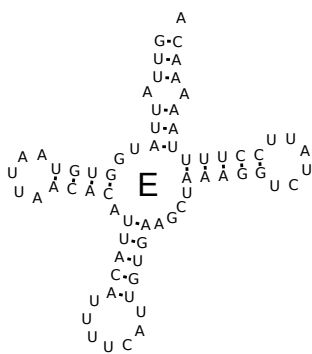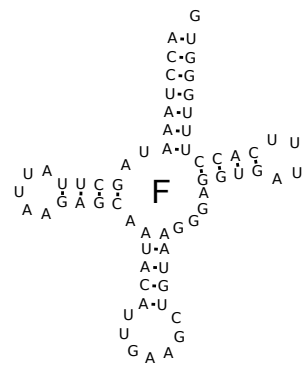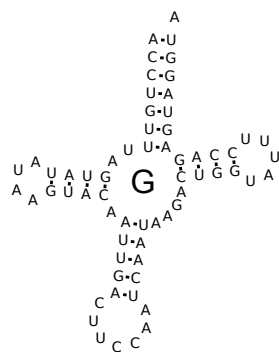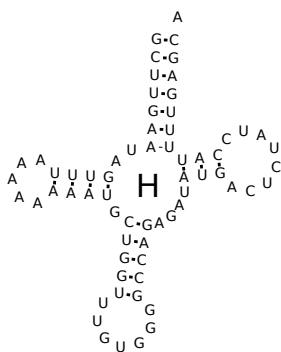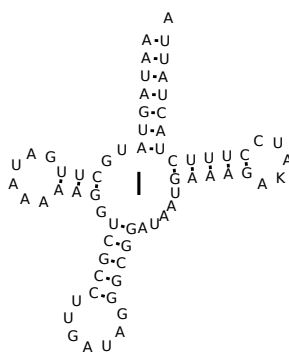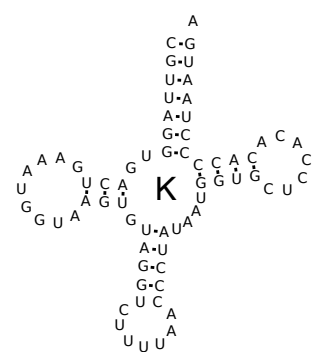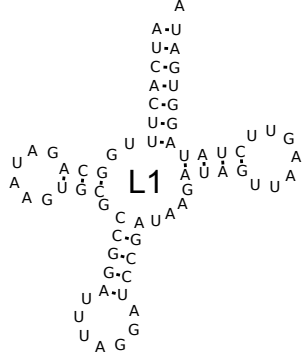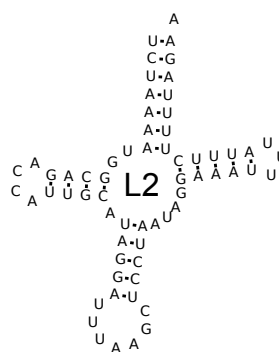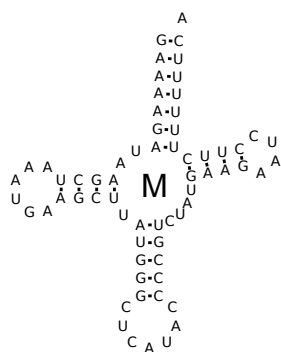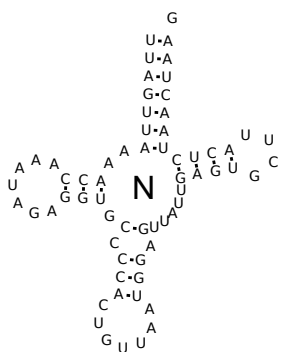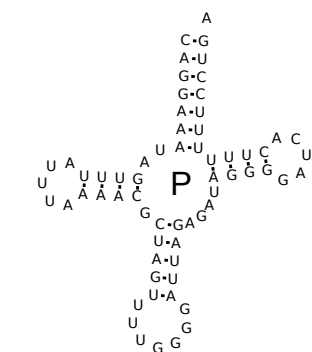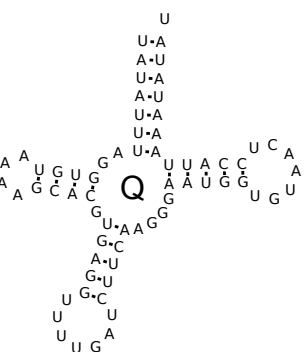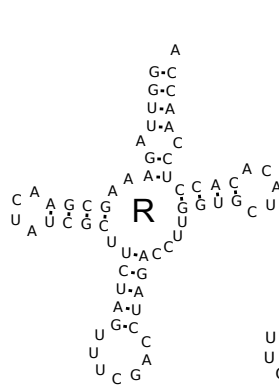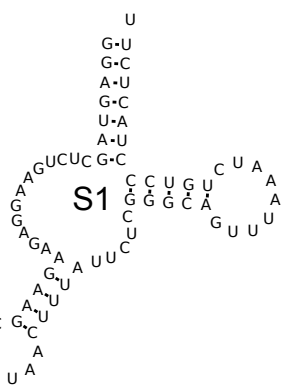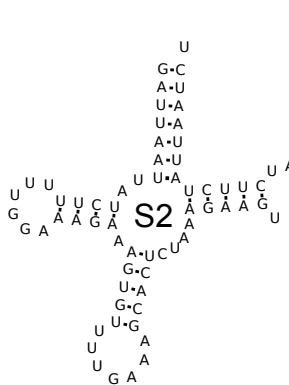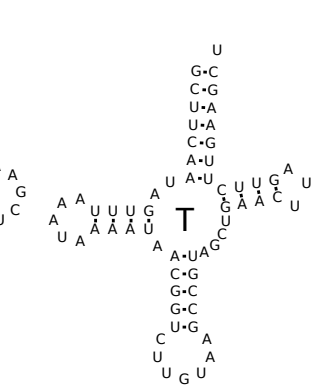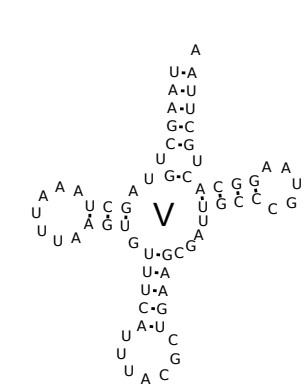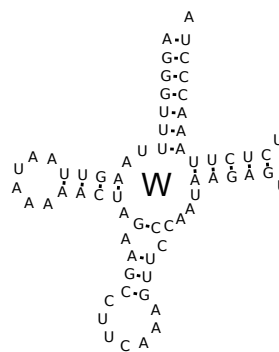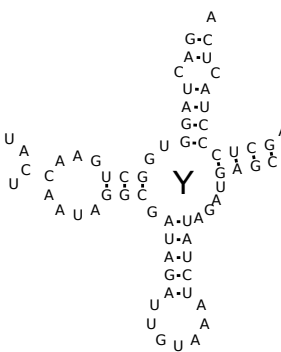

Supplement: S3 Fig — tRNAs are referred by single letter amino acid codes. (PDF) [file pone.0237037.s003.pdf]

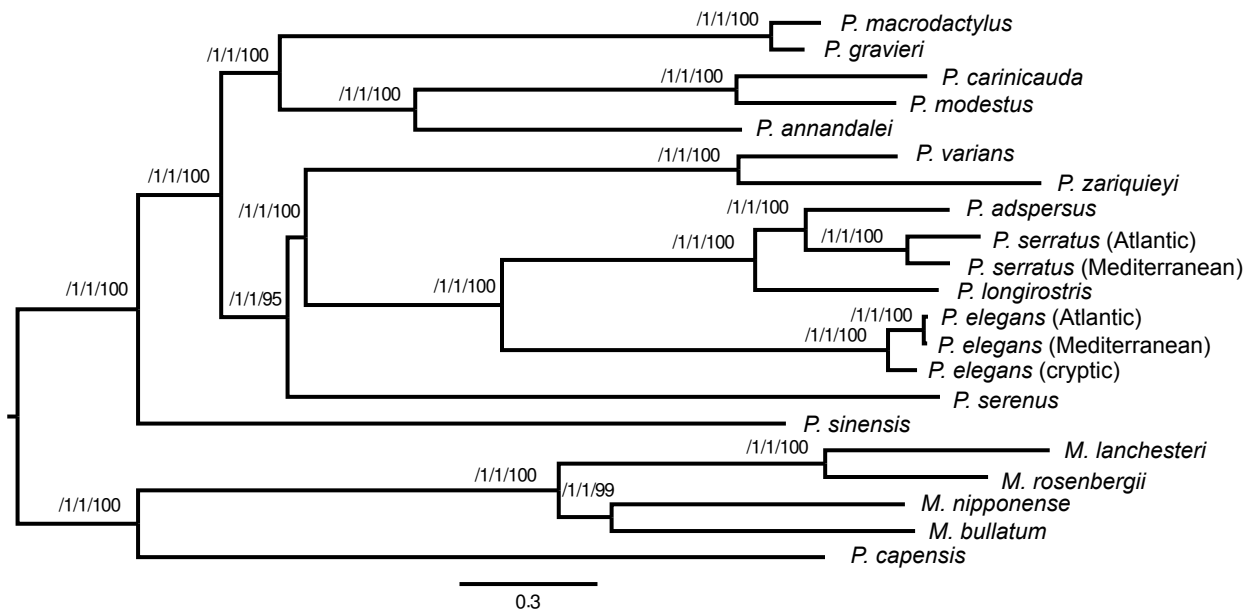

Supplement: S4 Fig — Numbers on nodes indicate support values for aBayes/aLRT/bootstrap. (PDF) [file pone.0237037.s004.pdf]
